# Exploring the Common Mutational Landscape in Cutaneous Melanoma and Pancreatic Cancer

**DOI:** 10.1111/pcmr.13210

**Published:** 2024-11-28

**Authors:** Elisabetta Broseghini, Federico Venturi, Giulia Veronesi, Biagio Scotti, Marina Migliori, Desy Marini, Claudio Ricci, Riccardo Casadei, Manuela Ferracin, Emi Dika

**Affiliations:** ^1^ IRCCS Azienda Ospedaliero‐Universitaria di Bologna Bologna Italy; ^2^ Department of Medical and Surgical Sciences (DIMEC) University of Bologna Bologna Italy; ^3^ Oncologic Dermatology Unit IRCCS Azienda Ospedaliero‐Universitaria di Bologna Bologna Italy; ^4^ Internal Medicine Unit IRCCS Azienda Ospedaliero‐Universitaria di Bologna Bologna Italy; ^5^ Pancreas and Endocrine Surgery Unit IRCCS Azienda Ospedaliero‐Universitaria di Bologna Bologna Italy

**Keywords:** cutaneous melanoma, genomics, germline mutations, pancreatic cancer

## Abstract

Cutaneous melanoma (CM) and pancreatic cancer are aggressive tumors whose incidences are rapidly increasing in the last years. This review aims to provide a complete and update description about mutational landscape in CM and pancreatic cancer, focusing on similarities of these two apparently so different tumors in terms of site, type of cell involved, and embryonic origin. The familial forms of CM and pancreatic cancers are often characterized by a common mutated gene, namely *CDKN2A*. In fact, a germline mutation in *CDKN2A* gene can be responsible for the development of the familial atypical multiple mole and melanoma syndrome (FAMMM), which is characterized by melanomas and pancreatic cancer development. Sporadic melanoma and pancreatic cancer showed different key‐driven genes. The open‐access resource cBioPortal has been explored to deepen and investigate the common mutational landscape of these two tumors. We investigated the common mutated genes found in both melanoma and pancreatic cancer with a frequency of at least 5% of tested patients and copy number alterations with a frequency of at least of 3%. Data showed that 18 mutated genes and 3 copy number alterations are present in both melanoma and pancreatic cancers types. Since we found two patients that developed both melanoma and pancreatic cancer, we compared mutation landscape between the two tumors and identified a pathogenic variant in *BRCA2* gene. This review gives valuable insights into the genetic underpinnings of melanoma and pancreatic cancer, urging the continued exploration and research of new genetic biomarkers able to identify patients and families at high risk of developing both cancers and to address to screening and to an effective clinical management of the patient.

**Summary**

- Familial melanoma and pancreatic cancer can be associated with an inheritance pattern of germline mutations in predisposing genes.
- This study contributes valuable insights to identify patients at high risk of developing both cancers and to guide clinicians in performing appropriate genetic testing and surveillance program.

## 1 Introduction

It is now recognized that familial melanoma and pancreatic cancer can be linked to inherited germline mutations in predisposing genes. This review gives valuable insights into the genetic underpinnings of melanoma and pancreatic cancer, urging the continued exploration and research of new genetic biomarkers able to identify patients and families at high risk of developing both cancers and to address such patients to screening programs for early identification of such cancers and to an effective clinical management of the patient to paving the way for possible future treatments based on gene‐targeted therapies (Ribero et al. 2016; Aung et al. 2018). To deepen and investigate the genetic landscape of these two cancers, an open‐access and open‐source resource, namely cBioPortal for Cancer Genomics, has been used to explore multidimensional cancer genomics datasets.

## 2 Germline Mutations in Cutaneous Melanoma and Pancreatic cancer

Cutaneous melanoma (CM) is a malignant tumor that arises from the transformation of pigment‐producing cells known as melanocytes. CM incidence has increased during the past several decades and, although CM accounts for 3%–5% of all skin cancers, it determines approximately 65% of all skin cancer deaths (Gershenwald and Scolyer 2018). The etiology of CM has been attributed mainly to environmental factors such as ultraviolet (UV) exposure. Other risk factors include patient‐related parameters such as fair phototypes and multiple dysplastic nevi. Genetic factors are less frequent; however, a family history of CM poses the highest risk for the development of melanoma (Dika, Broseghini, et al. 2021).

Around 7%–15% of CM cases occur in patients with a family history of CM; however, this does not automatically indicate that a single genetic mutation is being transmitted in those kindreds. In fact, the majority of family history of melanoma are due to sporadic cases related to shared UV exposures experiences, geographic location, and susceptible skin types. Only about 10% of patients with family history are classified as hereditary melanoma. Patients with a genetic predisposition often showed early onset and multiple primary melanomas (MPM) (Ribeiro Moura Brasil Arnaut et al. 2021). MPM refers to a patient that has developed at least two primary CMs in its lifetime. The highest risk to develop another melanoma is in the first year following the diagnosis of the primary melanoma, but the risk remains increased for at least 20 years (Dika, Broseghini, et al. 2021; Dika, Patrizi, et al. 2021; Zocchi et al. 2021; Lambertini et al. 2023).

Several genes have been identified as involved to melanoma predisposition, some of them are still with unknown clinical relevance (Ribeiro Moura Brasil Arnaut et al. 2021; Toussi et al. 2020). The most frequent germline mutations responsible for the development of melanoma are reported in Table 1.

**TABLE 1:** Germline mutation genes associated with melanoma.

Gene	Description—gene name	Protein	Specific function	Effect of mutations	Clinical phenotype	Prevalence of mutations	References
CDKN2A	Cyclin dependent kinase inhibitor 2A	p^16INK4a^	Inhibition of CDK4 and phosphorylation of RB	Loss‐of‐function, cell cycle progression	Superficial spreading melanoma. Pigmentation, pagetoid scatter, and spindle cell morphology in vertical growth phase	20%–40% of melanoma‐prone families	Ribeiro Moura Brasil Arnaut et al. (2021); Toussi et al. (2020); Rossi et al. (2019)
		p^14ARF^	Inhibition of HDM2 and ubiquitination of p53	Loss‐of‐function, cell cycle progression		~1% of melanoma‐prone families	Ribeiro Moura Brasil Arnaut et al. (2021); Toussi et al. (2020); Rossi et al. (2019)
CDK4	Cyclin‐dependent kinase 4	CDK4	Inhibition of p16 tumor suppressor leading to phosphorylation of RB and cell cycle progression	Alteration in binding domain, leading to reduced p16^INK4A^ inhibition and, to cell cycle progression	Superficial spreading melanoma. Pigmentation and pagetoid scatter	0.68%–1.4% of melanoma‐prone families	Ribeiro Moura Brasil Arnaut et al. (2021); Toussi et al. (2020); Potrony et al. (2015); Harland et al. (2016); Puntervoll et al. (2013)
BAP1	BRCA1 associated protein 1	BAP1	Deubiquitinating enzyme, BRCA1 binding partner Implicated in chromatin modulation, transcriptional regulation, and DNA damage repair	Loss‐of‐function, production of an altered protein that cannot function normally and may be broken down prematurely	BAP1‐inactivated nevi: Exclusively or predominantly intradermal Associated common nevus component often present (a combined nevus) Epithelioid melanocytes with round to oval vesicular nuclei and abundant amphophilic cytoplasm May show smaller epithelioid cells without abundant eosinophilic cytoplasm May show rhabdoid features	< 1% of melanoma‐prone families	Ribeiro Moura Brasil Arnaut et al. (2021); Toussi et al. (2020); Potrony et al. (2015); Aoude, Wadt, et al. (2015); Zhang et al. (2019); Kwon, Lee, and Lee (2023)
POT1	Protection of telomeres 1	POT1	Constituent of shelterin complex that regulates telomere processing and stability	Lead to longer telomeres that predispose individuals to cancer	Superficial spreading melanoma	0.48%–1.7% of melanoma‐prone families	Ribeiro Moura Brasil Arnaut et al. (2021); Toussi et al. (2020); Potrony et al. (2015); Robles‐Espinoza et al. (2014); Zade and Khattar (2023)
ACD	ACD shelterin complex subunit and telomerase recruitment factor	ACD	Constituent of shelterin complex that regulates telomere processing and stability	Lead to longer telomeres that predispose individuals to cancer	Superficial spreading melanoma, and lentigo maligna melanoma	< 1% of melanoma‐prone families	Ribeiro Moura Brasil Arnaut et al. (2021); Toussi et al. (2020); Potrony et al. (2015); Aoude, Wadt, et al. (2015); Aoude, Pritchard, et al. (2015)
TERT	Telomerase reverse transcriptase	Catalytic subunit of telomerase	Telomerase reverse transcriptase, a component of telomerase. Normally repressed in postnatal somatic cells leading to shortening of telomeres	Lead to longer telomeres that predispose individuals to cancer	Nodular and superficial spreading melanoma	0.04%–0.7% of melanoma‐prone families	Ribeiro Moura Brasil Arnaut et al. (2021); Toussi et al. (2020); Potrony et al. (2015); Harland et al. (2016)
TERF21P	TERF2 interacting protein	TERF21P	Constituent of shelterin complex that regulates telomere processing and stability	Lead to longer telomeres that predispose individuals to cancer	Superficial spreading melanoma, and lentigo maligna melanoma	< 1% of melanoma‐prone families	Ribeiro Moura Brasil Arnaut et al. (2021); Toussi et al. (2020); Potrony et al. (2015); Aoude, Wadt, et al. (2015); Aoude, Pritchard, et al. (2015)
MITF	Melanocyte inducing transcription factor	MITF	A melanocytic lineage‐specific transcription factor, regulating differentiation, proliferation and survival of melanocytes	Become a lineage‐survival oncogene	Amelanotic. Thicker tumors (in some populations). Nodular melanoma	1.6%–2.8% of melanoma patients	Ribeiro Moura Brasil Arnaut et al. (2021); Toussi et al. (2020); Yokoyama et al. (2011); Bertolotto et al. (2011); Ciccarese et al. (2020); Garraway and Sellers (2006)
MC1R	Melanocortin‐1 receptor	MC1R	G protein coupled receptor for melanocyte‐stimulating hormone. Controls melanogenesis, and skin and hair color	Loss‐of‐function, decreased eumelanin synthesis leading to fair skin and an increased sensitivity to UV exposure	Anatomic site (arms; in carriers of more than one high‐risk variant)	61%–66% of melanoma patients (at least one main MC1R variants)	Ribeiro Moura Brasil Arnaut et al. (2021); Toussi et al. (2020); Williams et al. (2011); Wolf Horrell, Boulanger, and D'Orazio (2016)

*Note:* Table was modified from Ribeiro Moura Brasil Arnaut et al. (2021) and Toussi et al. (2020).

More than 20% of familial CM cases are caused by a mutation in a high‐risk tumor predisposition gene, namely cyclin‐dependent kinase inhibitor 2A (*CDKN2A*). Germline mutations in *CDKN2A* increase the risk of melanoma by 65‐fold. In addition, the germline mutations in *CDKN2A* are present in 8%–15% of patients diagnosed with MPM without familial history and up to 40% in patients with familial melanoma (Helgadottir et al. 2017). Interestingly, the probability to found *CDKN2A* mutations is about 1% in sporadic melanoma patients without personal and/or familial history of melanoma (Harland et al. 2014).

Germline mutations in *CDKN2A* gene can be responsible for the development of a syndrome characterized by multiple clinically atypical nevi, melanomas and, in a subset of patients, pancreatic cancer. This melanoma‐related syndrome was first described by Lynch and Krush (1968) as familial atypical multiple mole and melanoma syndrome (FAMMM) and, second, by Clark Jr. et al. (1978) as B‐K mole syndrome or dysplastic nevus syndrome.

The connection between FAMMM and pancreatic cancer has been widely studied (Goldstein et al. 2006). In FAMMM patients, there is an estimated risk to develop pancreatic cancer 13–22 times higher than the risk in the average population, and the risk increases to more of 40‐fold in *CDKN2A*‐mutant FAMMM patients (Lynch et al. 2008; de Snoo et al. 2008). In melanoma prone families, pancreatic cancer was observed in 28% of *CDKN2A*‐mutant kindreds compared to only 6% of *CDKN2A*‐wild‐type kindreds. Moreover, 74% of families with pancreatic cancer harbored a *CDKN2A* mutation compared to 33% of those families with only melanoma (Goldstein et al. 2006). The penetrance for pancreatic cancer has been estimated as 17% in *CDKN2A* mutation carriers by 75 years of age (Vasen et al. 2000).

Studies have shown the presence of the inactivate protein product of *CDKN2A* gene, namely p^16INK4a^, in 95% of sporadic pancreatic cancers; however, germline *CDKN2A* mutations in these cases are rare (Bartsch et al. 2002).

Pancreatic cancer is one of the most aggressive tumors and its incidence and mortality are rapidly increasing. Only around 16% of patients have a resectable tumor at time of diagnosis. Around 94% of pancreatic cases develop in the exocrine tissue of the pancreas resulting in pancreatic ductal adenocarcinoma (PDAC). Other pancreatic cancer malignancies are rarer, such as (adeno)squamous carcinoma, colloid carcinoma, and neuroendocrine tumors (Klatte et al. 2022).

Pancreatic cancer is a multifactorial genetic disease. The general population has an average lifetime risk of pancreatic cancer of approximately 1.5%. Several high‐risk factors have been identified, such as smoking, daily alcohol consumption, and obesity (defined as BMI ≥ 30); and patients related nonmodifiable parameters such as older age, male gender, African‐Americans ethnicity, non‐O blood group, diabetes, chronic pancreatitis, and genetic mutations (Abe et al. 2021).

When the lifetime risk of pancreatic cancer is higher than 5%, individuals are considered high‐risk individuals (HRI). Up to 10% of pancreatic cancers cases arise in HRI, which can be stratified as familial pancreatic cancer, namely patients with a strong family history, or as hereditary cancer syndrome, namely patients that are carriers of a germline mutation (Klatte et al. 2022). Pancreatic cancer is associated with both family and hereditary cancer syndromes (Abe et al. 2021).

Familial pancreatic cancer indicates a family clustering of pancreatic cancer with two or more first‐degree relatives with pancreatic cancer and without a known hereditary cancer syndrome. The risk to develop a pancreatic cancer increases with the number of affected family members: from a 4.6‐fold risk with one first‐degree relative to 32‐fold risk with three first‐degree relatives (Klatte et al. 2022). There are known germline pathogenic variants linked to hereditary pancreatic cancer, which is closely related to other hereditary tumor syndromes (Abe et al. 2021; Table 2).

**TABLE 2:** Hereditary tumor syndromes associated with pancreatic tumor.

Cancer syndrome	Relative risk for pancreatic cancer	Most frequent associated cancers	Gene	Description—gene name	Role	Specific function	References
Familial Atypical Multiple Melanoma Mole (FAMMM) syndrome	13–47.8	Melanoma and non‐melanoma skin, oropharynx, respiratory	CDKN2A (p^16INK4a^)	Cyclin‐dependent kinase inhibitor 2A	Cell cycle	Deceleration of cell cycle by inhibition of CDK4 and phosphorylation of RB	de Snoo et al. (2008); Helgadottir et al. (2020)
Familial Adenomatous Polyposis (FAP)	4.5	Gastrointestinal, brain, thyroid, hepatic	APC	Adenomatous Polyposis Coli	Proliferation	Inhibition of Wnt signaling pathway	Vasen et al. (2008)
Ataxia‐telangiectasia (AT)	6.5	Lymphoma, acute leukemia, breast	ATM	ataxia telangiectasia mutated	DNA damage responses	Regulation of double‐strand DNA break (DSB) signaling and stress responses	Hsu et al. (2021)
Hereditary Breast and Ovarian syndrome (HBOC)	2.3–10.0	Breast, ovarian, prostate	BRCA1 BRCA2	Breast Cancer susceptibility gene 1/2	Genome stability	Regulation of precise DNA repair by homologous recombination	Nelson et al. (2013); van Asperen et al. (2005)
Peutz‐Jeghers syndrome (PJS)	132	Colorectal, breast, small bowel, gastric	STK11 (known also as LKB1)	Serine/Threonine Kinase 11	Cell polarity and energy metabolism	STK11: Control of the activity of AMP‐activated protein kinase (AMPK) with the formation of heterotrimeric complex	van Lier et al. (2010); Giardiello et al. (2000)
Hereditary nonpolyposis colorectal cancer (Lynch syndrome)	8.6	Gastrointestinal, endometrial, urological	MLH1 MSH2 MSH6	MutL homolog 1 DNA mismatch repair protein Msh2/6	DNA mismatch repair	Proteins responsible of DNA mismatch repair	van Leerdam et al. (2019); Kastrinos et al. (2009); Watson et al. (2008)
Li‐Fraumeni syndrome	7.3	Breast, sarcoma, leukemia, adrenocortical, brain	TP53 (p53)	Tumor protein P53	Apoptosis	Promotion of apoptosis after detection of DNA damage	de Andrade et al. (2019); Ruijs et al. (2010)

*Note:* Table was modified from Klatte et al. (2022).

In addition of the accurate described *CDKN2A* gene (Danishevich et al. 2023; Pauley et al. 2022), there are other genes found in literature that are associated with both melanoma and pancreatic cancers. Some genes responsible of tumor syndromes listed in Table 2 were also found mutated in melanoma patients. For example, a likely pathogenic mutation of the Peutz‐Jeghers syndrome gene, namely *STK11*, were found in primary melanoma (Rowan et al. 1999). STK11 can prevent melanoma cell invasion through the activation of the signal transducer and activator of STAT3/5 and FAK signaling pathways (Dzung et al. 2022; Azin and Demehri 2022). Malignant melanoma has been reported also in *BRCA2*‐mutated families (Johansson et al. 2019). Heterozygous and loss‐of‐function germline variants in *ATM* gene have been also associated with an increased lifetime risk of melanoma cancers (Borja et al. 2023).

## 3 Somatic Alterations in Cutaneous Melanoma and Pancreatic cancer

Despite family and hereditary cases, the majority of melanoma and pancreatic cancer cases are attributable to randomly acquired genetic mutations, which can contribute to cancer development and metastasis progression. It is essential to identify the somatic driver mutations in tumors, because they play a critical role in early diagnosis, optimal management, precise prognostication and targeted therapy approaches.

In melanoma, several molecular factors, including non‐coding RNA and somatic mutations, have been associated with melanoma pathogenesis (Ribero et al. 2023; Durante et al. 2022, 2021; Dika et al. 2020; Riefolo et al. 2019; Broseghini et al. 2021; Naddeo et al. 2024). Moreover, several genes have been found implicated in the activation of the MAPK pathway, affecting cell proliferation, differentiation and survival, and in PI3K/Akt pathway, affecting metabolism, proliferation, cell survival, growth and angiogenesis (Dika, Patrizi, et al. 2020). A genomic classification of melanoma is based on the presence or absence of somatic alterations of three genes. Specifically, melanomas can be classified into four genomic subtypes based on the pattern of the most prevalent significantly mutated genes: mutant BRAF, mutant RAS (N‐, H‐, K‐), mutant NF1, and Triple‐wild type (Triple‐WT). The Triple‐WT group is enriched of KIT mutations, focal amplifications, and complex structural rearrangements. In addition, it lacks UV signature and manifests more copy number changes (Cancer Genome Atlas Network 2015). Other somatic mutations have been described in literature (Ravaioli et al. 2019; Lambertini, Mussi, and Dika 2021; Moscarella et al. 2019; Querzoli et al. 2023; Scarfi et al. 2020; Starace et al. 2018); Table 3 reports the most significant somatic driver mutations within the subtypes of primary melanoma, namely nonacral CM, which includes superficial spreading melanoma (SSM), nodular melanoma (NM), and lentigo maligna melanoma (LMM); desmoplastic melanoma, acral lentiginous melanoma (ALM), and mucosal melanoma.

**TABLE 3:** Most significant somatic driver mutations in cutaneous melanoma.

Gene	Description—gene name	Pathway	Protein type	Most frequent mutations‐alterations	Clinical phenotype	Prevalence of mutations	Consequences due to mutation	References
BRAF	B‐Raf Proto‐Oncogene	MAPK pathway	Serine/threonine protein kinase	V600E, V600K, V600D, V600R; mutually exclusive of NRAS mutations	Nonacral cutaneous melanoma, younger patients, sites with intermittent sun exposure	60% of melanomas	Constitutive activation of MAPK signaling	Hawryluk and Tsao (2014); Manzano et al. (2016)
NRAS	Neuroblastoma RAS viral oncogene homolog	MAPK pathway	G‐regulatory protein	Q61K, Q61R, Q61L; mutually exclusive of BRAF mutations	Nonacral cutaneous melanoma, non‐sun‐damaged skin regions, increased Breslow thickness, higher mitotic rates, lower incidence of ulceration	15%–30% of melanomas	Constitutive activation of MAPK signaling	Cancer Genome Atlas Network (2015); Hawryluk and Tsao (2014); Manzano et al. (2016); Johnson and Puzanov (2015)
NF1	Neurofibromin 1	MAPK pathway and PI3K pathways	Negative regulator of RAS	Loss‐of‐function mutation; coexisted with mutations of RASA1	Desmoplastic melanomas, sun‐exposed melanomas and elderly patients	14% of melanomas	Constitutive activation of the MAPK and PI3K pathways	Cancer Genome Atlas Network (2015); Manzano et al. (2016); Kadokura et al. (2016)
KIT	Proto‐oncogene receptor tyrosine kinase	MAPK and PI3K/Akt pathways	Transmembrane receptor tyrosine kinase	Exon 11 (most often L576P), exon 13 (most often K642E); mutually exclusive of BRAF and NRAS mutations	Acral lentigo melanoma, mucosal melanoma, chronically sun‐damaged surfaces	3% of all melanomas	Induction of both the MAPK and the PI3K pathways	Carvajal et al. (2015); Curtin et al. (2006)
PTEN	Phosphatase and Tensin homolog	PI3K/Akt pathway	Phosphatidyl‐inositol‐3,4,5‐triphosphate 3‐phosphatase	Frameshift mutation, chromosomal deletion; frequently coexist with BRAF mutations, but not with NRAS	Nonacral cutaneous melanoma	14% of specimens in the TCGA melanoma cohort	Activation of PI3K/Akt pathway	Cancer Genome Atlas Network (2015); Shain et al. (2015)
MITF	Microphthalmia‐associated transcription factor	Melanogenesis, MAPK pathway	Basic helix–loop–helix/leucine zipper transcription factor	M‐MITF isoform, MITF amplification	Nonacral cutaneous melanoma	80% of melanomas 20% of metastatic melanomas	Promotion tumorigenesis	Garraway et al. (2005); Wellbrock and Arozarena (2015)
PREX2	Phosphatidylinositol‐3,4,5‐trisphosphate RAC exchanger 2	PI3K/AKT pathway	Protein that activates the small GTPase Rac	Truncated mutation	Nonacral cutaneous melanoma	27% of the TCGA melanoma cohort	Acceleration of tumor formation, activate RAC1 increased PI3K/AKT signaling, enhanced cell proliferation	Lissanu Deribe (2016); Berger et al. (2012)
TERT	Telomerase reverse transcriptase	Telomere sustainment, chromosomal stability	Catalytic subunit of the holoenzyme telomerase	C228T, C250T (ultraviolet‐signature mutation) TERT promoter mutations are associated with BRAF and NRAS mutations	Nonacral cutaneous melanoma	Sporadic primary melanomas (33%), metastatic melanomas (85%)	Overcome replication‐induced senescence	Huang et al. (2013); Horn et al. (2013); Griewank et al. (2014)
RAC1	Rac Family Small GTPase 1	Cellular cytoskeleton organization and motility	RHO GTPase	P29S (ultraviolet‐signature mutation)	Nonacral cutaneous melanoma, increased Breslow thickness, increased mitotic rate, ulceration, presence of lymph node metastases at the time of diagnosis	5%–9% of melanomas	Cell proliferation, suppression of antitumor immune responses	Vu et al. (2015); Mar et al. (2014)
RPS27	Protein S27	Translation regulation		5′‐UTR mutation	Nonacral cutaneous melanoma	10% of melanomas	Induction of the mTOR pathway	Dutton‐Regester et al. (2014)
CCND1	Cyclin D1	Cell cycle	Cell cycle control protein	Amplification	Acral lentigo melanoma, superficial spreading melanoma	44% of ALM and 6% of SSM	Promotion of cell cycle entry	Sauter et al. (2002)

About 90% of PDCA occur in sporadic forms and clinical heterogeneity is a hallmark of this disease (Morani et al. 2020; Pompella et al. 2020). In addition, the genomic analysis has always been very difficult, since PDAC often contains only 5%–20% of neoplastic cells and cancers with lower cellularity than 40% often have been excluded (Waddell et al. 2015). Nevertheless, genomic researches revealed four key driver genes that are characteristically found in most PDACs, namely *KRAS*, *CDKN2A*, *TP53*, and *SMAD4* (Morani et al. 2020; Pompella et al. 2020). Specifically, PDACs present significantly recurrent mutations, namely gain‐of‐function mutation in *KRAS* oncogene; loss‐of‐function mutations in *CDKN2A*, *TP53* and *SMAD4* tumor suppressor genes (Pompella et al. 2020).

Also, other chromosomal alterations have been described, and specific genetic mutations are associated with characteristic subtypes of PDACs and their precursors (Table 4). The precursor of the majority of PDACs is pancreatic intraepithelial neoplasia (PanIN), while only a minority arises from pancreatic cystic lesions, namely intraductal papillary mucinous neoplasms (IPMNs) and mucinous cystic neoplasms (Pompella et al. 2020). *KRAS* somatic mutations are frequently found also in low‐grade PanINs, while high‐grade PanINs display *CDKN2A* and *TP53* mutations, too. *TP53* and *SMAD4* inactivation seems to be the final events of the molecular cascade that lead to the transformation in PDAC. IPMNs are characterized especially by mutations in *GNAS* and *KRAS*. Similar to PanINs, *TP53* and *SMAD4* gene mutations could be seen as the latest molecular events to the PCDA transformation (Pompella et al. 2020).

**TABLE 4:** Most significant somatic driver mutations in pancreatic cancer.

Gene	Description—gene name	Pathway	Protein type	Most frequent mutations‐alterations	Clinical phenotype	Prevalence of mutations in pancreatic adenocarcinoma	Consequences due to mutation	References
KRAS	Kirsten rat sarcoma viral oncogene homolog	MAPK signaling	Small GTPase transductor protein	Missense mutation, exons 2 and 3	Pancreatic Ductal Adeno Carcinoma; Intraductal papillary mucinous neoplasm; Pancreatic intraepithelial neoplasms	70%–95%	Constitutively activation of KRAS and MAPK signaling	Cicenas et al. (2017); Hayashi et al. (2017); Wood, Yurgelun, and Goggins (2019)
CDKN2A	Cyclin Dependent Kinase Inhibitor 2A	Cell cycle control	Cyclin‐dependent kinase inhibitor	Inactivating mutation/LOH, homozygous deletion	Pancreatic Ductal Adeno Carcinoma; Intraductal papillary mucinous neoplasm; Pancreatic intraepithelial neoplasms	49%–98%	Alteration of cell cycle	Cicenas et al. (2017); Hayashi et al. (2017); Luo et al. (2013)
TP53	Tumor protein P53	DNA damage response	Regulatory protein	Missense mutation/LOH	Pancreatic Ductal Adeno Carcinoma; Intraductal papillary mucinous neoplasm; Pancreatic intraepithelial neoplasms	20%–76%	Loss of DNA binding ability and gene transcription activation, loss of apoptosis	Cicenas et al. (2017); Hayashi et al. (2017); Wood, Yurgelun, and Goggins (2019)
SMAD4	SMAD family member 4	TGFβ signaling	Signal transduction proteins	Inactivating mutation/LOH, homozygous deletion	Pancreatic Ductal Adeno Carcinoma	19%–50%	Alteration of TGFβ pathway	Cicenas et al. (2017); Hayashi et al. (2017); Wood, Yurgelun, and Goggins (2019); De Bosscher, Hill, and Nicolas (2004)
GNAS	Guanine Nucleotide binding protein Alpha Stimulating activity polypeptide	G‐protein signaling	Component of G protein‐coupled receptor‐regulated adenylyl cyclase signal transduction pathways	Missense mutation	Pancreatic Ductal Adeno Carcinoma; Intraductal papillary mucinous neoplasm	2%–11%	Induction of PKA‐mediated SIK suppression and reprogramming lipid metabolism	Wood, Yurgelun, and Goggins (2019); Patra et al. (2018)
BRCA1	Breast cancer type 1 susceptibility protein	DNA repair	Nuclear phosphoprotein	Inactivating mutation/LOH	Pancreatic Ductal Adeno Carcinoma	6.6%–14%	Alteration in DNA repair	Cicenas et al. (2017); Wood, Yurgelun, and Goggins (2019); Stadler et al. (2012); Knudsen et al. (2016)
BRCA2	Breast cancer type 2 susceptibility protein	DNA repair	Nuclear phosphoprotein	Inactivating mutation/LOH	Pancreatic Ductal Adeno Carcinoma	3.6%–7.5%	Alteration in DNA repair	Cicenas et al. (2017); Stadler et al. (2012); Friedenson (2005)
RNF43	Ring finger protein 43	Ubiquitin signaling	Ubiquitin‐protein ligase	Inactivating mutation/LOH	Pancreatic Ductal Adeno Carcinoma; Intraductal papillary mucinous neoplasm	10%–15%	Induction of constitutive activation of Wnt signaling	Wood, Yurgelun, and Goggins (2019)
ARID1A	AT‐rich interaction domain 1A	Chromatin remodeling	Subunit of SWI/SNF protein complexes	Inactivating mutation/LOH	Pancreatic Ductal Adeno Carcinoma	10%–25%	Disruption of the f SWI/SNF chromatin remodeling complex	Wood, Yurgelun, and Goggins (2019); Witkiewicz et al. (2015)
SLIT2	Slit Guidance Ligand 2	Axon guidance		Mutation/LOH	Pancreatic Ductal Adeno Carcinoma	5%–15% o	Regulation of tumor angiogenesis, cell invasion, metastasis, and nerve infiltration	Wood, Yurgelun, and Goggins (2019); Ding et al. (2020); Secq et al. (2015)
MYC	Myelocytomatosis oncogene	Transcriptional regulation	Nuclear phosphoprotein	Amplification	Pancreatic Ductal Adeno Carcinoma	10%–15%	Tumor promotion	Wood, Yurgelun, and Goggins (2019)

As reported, sporadic CM and sporadic pancreatic cancer seem to be characterized by different key‐driven genes. Interestingly, genetic alterations in *CDKN2A* gene are frequently found in both familial and sporadic pancreatic cancer (Tables 2 and 4) while in melanoma, this gene is mostly associated with the familial form (Table 1). These data suggested that the link between these two tumors is mostly at germline mutation levels.

## 4 Open‐Access Cancer Genomics Analysis in Melanoma and Pancreatic Cancer

To deepen and investigate the genetic landscape of these two cancers, an open‐access and open‐source resource, namely cBioPortal for Cancer Genomics (https://genie.cbioportal.org), has been used to explore multidimensional cancer genomics datasets. Specifically, the last AACR Project GENIE registry (GENIE 15.0‐public, which was released in January 2024) has been used and investigated. This registry is one of the largest fully public cancer genomic datasets released to date, which includes approximately 198,000 sequenced samples from more than 172,000 patients (https://www.aacr.org/professionals/research/aacr‐project‐genie/aacr‐project‐genie‐data/).

Among the 198,041 samples, we selected and analyzed (March 1, 2024) melanoma samples (melanoma *n* = 4218, CM *n* = 2193) and pancreatic cancer samples (pancreatic adenocarcinoma *n* = 7261, pancreatic neuroendocrine tumor *n* = 689, adenosquamous carcinoma of the pancreas *n* = 123). For both tumor types, we collected mutated genes with a frequency ≥ 5% and copy number alterations (CNA) with a frequency ≥ 3%.

For melanoma, 253 mutated genes with a frequency ≥ 5% were found and 169 of them were found in more than 1000 samples (Table S1). Fifteen mutated genes are highly frequent (frequency > 20%) in melanomas and include the three genes used for genetic classification of melanoma, namely *BRAF* (frequency = 38.4%), *NRAS* (frequency = 24.8%), and *NF1* (frequency = 21.8%). The other genes are *TERT* (frequency = 50.3%), *LRP1B* (frequency = 34.7%), *PTPRT* (frequency = 29.4%), *SPTA1* (frequency = 25.0%), *MECOM* (frequency = 24.6%), *GRIN2A* (frequency = 24.0%), *PREX2* (frequency = 24.0%), *KMT2D* (frequency = 22.7%), *PTPRD* (frequency = 21.8%), *GLI2* (frequency = 20.3%), *ROS1* (frequency = 20.1%), and *PAK5* (frequency = 20.0%).

For pancreatic cancer, we found 27 mutated genes with a frequency ≥ 5% (Table S2). Among them, 12 have been profiled in more than 1000 samples. The four most frequent mutations are those confirmed in sporadic pancreatic cancer by literature, namely *KRAS* (frequency = 78.9%), *TP53* (frequency = 64.0%), *SMAD4* (frequency = 18.2%) and *CDKN2A* (frequency = 9.4%). Other frequent mutations occur in already described *ARID1A* gene (frequency = 8.3%) and *RNF43* (frequency = 5.9%). Interestingly, *LRP1B* (low‐density lipoprotein receptor‐related protein 1B) gene has a frequency of 9.0%.

We compared the mutated genes in melanoma and pancreatic cancer, and we reported the 18 common mutated genes in Table 5, where number of profiled samples and frequency of the mutated gene are indicated in both tumors.

**TABLE 5:** Common mutated genes in melanoma and pancreatic cancer.

Gene	Description—gene name	Pathway	Melanoma		Pancreatic cancer	
			Frequency	Profiled samples (*n*)	Frequency	Profiled samples (*n*)
ADGRL3	Adhesion G Protein‐coupled receptor L3	Regulator of synaptic function	5.3%	57	5.5%	55
ANKRD24	Ankyrin repeat domain 24	Unknown molecular role	5.4%	56	13.0%	54
ARID1A	Chromatin remodeling gene AT‐rich interactive domain 1A	SWI/SNF complex, chromatin regulator	10.7%	4269	8.3%	7303
BOD1L1	Biorientation of chromosomes in cell division 1 like 1	Replication fork protection factor	7.1%	56	11.1%	54
CDH23	Cadherin‐23	Mediator of homotypic and heterotypic cell–cell adhesions	12.7%	63	15.6%	77
CDKN2A	Cyclin‐dependent kinase inhibitor 2A	Cell cycle	9.4%	6222	9.4%	8041
DNAH9	Dynein axonemal heavy chain 9	Cell–cell adhesion	16.7%	60	17.9%	84
KDM6B	Lysine demethylase 6B	Epigenetic modifier	8.5%	246	5.4%	410
KMT2D	Lysine methyltransferase 2D	H3K4 methylation	22.7%	4110	7.0%	7127
LAMB4	Laminin subunit beta 4	Constituent of the extracellular matrix	5.6%	72	5.4%	147
LRP1B	Low‐density lipoprotein receptor‐related protein 1B	LDL receptor family signaling	34.7%	1470	9.0%	1267
PDE4DIP	Phosphodiesterase 4D interacting protein	Endoplasmic reticulum‐to‐Golgi traffic	6.2%	113	5.7%	209
PKD1L2	Polycystin 1 Like 2 (gene/pseudogene)	Probably function as a G‐protein‐coupled component or regulator of cation channel pores	10.7%	56	14.8%	54
PRKDC	Protein kinase, DNA‐activated, catalytic subunit	DNA repair	14.5%	2526	5.5%	2934
RANBP2	Ran‐binding protein 2	Nuclear pore complex	10.2%	1015	5.4%	958
SPTA1	Spectrin alpha, erythrocytic 1	cytoskeletal protein	25.0%	1407	5.8%	1191
TGF‐β1	Transforming growth factor beta 1	Activation of SMAD family transcription factors	7.1%	56	5.6%	54
TP53	Tumor protein P53	DNA damage response	18.8%	6356	64.0%	8046

In addition to the thoroughly described *CDKN2A*, which has been found in melanoma and pancreatic cancer with the same frequency (9.4%) in AACR Project GENIE registry, data showed other interestingly and relevant genes, since they were profiled in many samples (more than 900 samples for both groups) and found highly mutated.

First, there is *TP53* that is one of the most frequently mutated gene in cancer. The inactivation of the gene leads to the loss of the tumor suppressor action of the protein and can show additional oncogenic functions that give growth and survival advantages. More than 60% of pancreatic cancer cases present at least one mutation in these gene. These data are in agreement with the literature, where *TP53* is described as one of the four most mutated genes in sporadic pancreatic cancer (Cicenas et al. 2017; Wood, Yurgelun, and Goggins 2019). In AACR Project GENIE registry, 18.8% of melanomas showed at least a mutation in *TP53*. In literature, it was observed that there is a higher prevalence of mutant p53 in metastatic melanoma compared to primary tumors (Khan et al. 2023; Ricci et al. 2020). Mutated *TP53* is also responsible for a mixed cancer syndrome, namely Li‐Fraumeni syndrome, which leads to develop several different types of cancers, including melanoma (Toussi et al. 2020).

Another gene that is often mutated in several cancers is *LRP1B*, specifically, this gene is frequently inactivated at both genetic and epigenetic levels. *LRP1B* encodes for a cell surface receptor and is broadly expressed in multiple normal tissues. *LRP1B* is a member of LDLR protein family with a wide range of biological functions from cargo transport to cell signaling (Principe et al. 2021). Data from Genie registry showed that *LRP1B* is frequently mutated in both melanoma samples (34.7%) and pancreatic cancer cases (9%). Inactivation of this gene was also reported in melanoma (Nikolaev et al. 2011) and some single nucleotide polymorphism have been significantly associated with pancreas cancer risk in literature (Cotterchio et al. 2015).

27% of melanomas and 7% of pancreatic cancers in GENIE cohort showed mutation in *KMT2D* gene, also known as mixed‐lineage leukemia protein 4 (*MLL4*), which is one of the most frequently mutated genes in human cancer. KMT2D is a highly conserved catalytic component of the mammalian complex of protein associated with Set1 (COMPASS) complex, and is responsible for catalyzing H3K4me1 at transcription enhancers throughout the human genome (Lin‐Shiao et al. 2018). *KMT2D* mutant melanomas have been described in literature, where it was found that *KMT2D* loss promotes tumorigenesis by facilitating an increased use of the glycolysis pathway (Murugesan and Maitituoheti 2021). High mutation rates of KMT2D have been reported also in pancreatic cancer (Froimchuk, Jang, and Ge 2017). However, the prognostic role of *KMT2D* in pancreatic cancers is ambiguous, with contrasting reported in different studies (Sausen et al. 2015; Dawkins et al. 2016; Koutsioumpa et al. 2019).

*SPTA1* gene encodes a cytoskeletal protein that affects cell and tissue growth and development by regulating YAP, an effector on the Hippo signaling pathway. This gene is mostly known as associated with Hereditary spherocytosis. Mutations on this gene were found in both pancreatic cancer, where it served as independent prognostic factor (Kou et al. 2023) and melanoma (Zhou et al. 2021). However, data about role of this gene in cancer are still poor, although they are frequently mutations in this gene in GENIE cohort of melanoma (25%) and pancreatic cancer (5.8%) samples.

Other four genes showed a frequency between 5% and 15% in a large number of tested melanoma and pancreatic cancer samples, namely *ARID1A*, *PDE4DIP*, *PRKDC*, and *RANBP2*.

ARID1A belongs to a class of chromatin regulatory proteins and was found mutated in both melanoma cases (10.7%) and pancreatic cancer samples (8.3%) in AACR Project GENIE registry. *ARID1A* role in cancer is highly variable since its alterations can have a tumor suppressive or oncogenic role, depending on the tumor type and context. ARID1A loss is generally associated with disease progression more often than onset (Fontana et al. 2023). In melanoma, *ARID1A* mutations are distributed across the gene without clustering or hotspots, and *ARID1A‐*mutated melanomas exhibit higher tumor mutational burden (TMB) compared to *ARID1A* wild‐type melanomas (Thielmann et al. 2022). In pancreatic ductal adenocarcinoma, *ARID1A* and *KRAS* have been found as co‐mutated, suggesting that the inactivation of ARID1A may cooperate with KRAS in the early stages of pancreatic cancer formation (Birnbaum et al. 2011).

*PDE4DIP* also known as myomegalin (*MMGL*) is a component of AKAP‐PKA‐PDE4D signaling complex, which anchors the sequestering components of the cAMP‐dependent pathway to Golgi and/or centrosomes. Somatic mutations in *PDE4DIP* gene have been found in some cancers, including melanoma and pancreatic cancer; however, *PDE4DIP* literature is still poor (Li et al. 2023). In AACR Project GENIE registry, the frequency is between 5% and 6% for both melanoma and pancreatic cancer.

*PRKDC* mutations have been detected in a high portion of melanoma samples (14.5%) and in 5.5% of tested pancreatic cancers. This gene encodes a protein that plays an important role in nonhomologous end joining (NHEJ) of DNA double‐strand breaks (DSBs) and is also closely related to the establishment of central immune tolerance and the maintenance of chromosome stability. There are emerging functions of this gene in the initiation and progression of cancer. Interestingly, the silencing of the *PRKDC* product by microRNAs sensitizes tumor cells to gemcitabine in pancreatic cancer cells (Hu et al. 2017) and cisplatin in melanoma cells (Li et al. 2017), respectively.

The nucleoporin *RANBP2* is a key component of the nuclear pore complex that regulates the nucleocytoplasmic transport. In GENIE cohort, this gene has been found mutated in 10.2% of melanoma samples and in 5.4% pancreatic cancer samples. Both tumor suppressor and oncogenic role have been described for *RANBP2* in cancer. A rare form of melanoma is the primary malignant melanoma of the esophagus (PMME). In this tumor, *RANBP2* gene has been found recurrently mutated and is considered one of the driver mutated genes. Moreover, all *RANBP2* mutations were putatively deleterious (Li et al. 2022).

Finally, other mutations have been found localized in several genes in a smaller number of tested melanoma and pancreatic cancer samples (*n* < 200), including ADGRL3, ANKRD24, BOD1L1, CDH23, DNAH9, KDM6B, LAMB4, PKD1L2, and TGF‐β1. Literature about ADGRL3, ANKRD24, BOD1L1, LAMB4, and PKD1L2 in melanoma and in pancreatic cancer is still missing, while the other genes of this list have been already studied in these two tumors.

Through high‐throughput sequencing, *CDH23* has been identified as a promising susceptibility gene in hereditary melanoma. Evaluating its expression in sporadic melanoma by using the TCGA dataset, *CDH23* was found downregulated and its loss was associated with worse survival (Campos et al. 2020). In pancreatic cancer, findings suggest that overexpression of *CDH23* is likely to be related to the PDCA biological aggressiveness (Taniuchi et al. 2005). *DNAH9* product is involved in cell‐to‐cell adhesion and markedly affects cancer cell invasion and migration. *DNAH9* gene has been used in a support vector machine signature, which can predict relapse and survival in patients with early‐stage pancreatic ductal adenocarcinoma (Huang et al. 2023). *KDM6B* belongs to lysine demethylases (KDMs) family that removes methyl groups on lysine (K) amino acids of histones. The aberrant KDMs’ activity affects epigenetic modifications and is found in a variety of cancers, including pancreatic cancer and melanoma. In pancreatic cancer, loss of *KDM6B* enhances aggressiveness of pancreatic cancer (Yamamoto et al. 2014). In melanoma, *KDM6B* activity leads to upregulation of various targets of both NF‐κB and BMP that results in promotion of cell migration and metastasis development (Karami Fath et al. 2022). In addition, *KDM6B* expression has been found as positive associated with immune infiltration level of Treg in both pancreatic cancer and melanoma (Ding et al. 2022). *TGF‐β1* plays a complex role in carcinogenesis, including in melanoma. In fact, increased circulating TGF‐β1 is associated with impairment in NK cell effector functions in metastatic melanoma patients (Mirjacic Martinovic et al. 2022). In pancreatic cancer, *TGF‐β1* promotes autophagy that promoted proliferation and inhibited migration in SMAD4‐positive PDAC cells by decreasing the nuclear translocation of SMAD4, while it inhibited proliferation and promoted migration in SMAD4‐negative cells through the regulation of MAPK/ERK activation (Liang et al. 2020).

To further investigate genes involved in the pathogenesis of melanoma and pancreatic cancer, we searched for patients who had both cancers in AACR Project GENIE registry. We found two patients: the first patient with the ID code GENIE‐DFCI‐202272 (https://genie.cbioportal.org/patient/summary?studyId=genie_public&caseId=GENIE‐DFCI‐202272) develops a primary pancreatic adenocarcinoma (GENIE‐DFCI‐202272‐2710284) and melanoma metastasis (GENIE‐DFCI‐202272‐4192215), while the second patient with ID code GENIE‐DFCI‐089908 (https://genie.cbioportal.org/patient/summary?studyId=genie_public&caseId=GENIE‐DFCI‐089908) develops a primary melanoma (GENIE‐DFCI‐089908‐282415) and presents a metastasis form pancreatic adenocarcinoma (GENIE‐DFCI‐089908‐2970125). In both patients, we focused and reported mutated genes present in both tumors (Table 6), because there might be some germline mutations that could predispose to melanoma and pancreatic cancer development.

**TABLE 6:** Common mutated genes in pancreatic cancer and melanoma from the same patient.

Patient	Gene	Name	Pathway/role	Mutation type	HGVSg^a^	Protein change	ClinVar (ClinVar ID)
GENIE‐DFCI‐202272 (primary pancreatic adenocarcinoma and melanoma metastasis)	BRCA2	Breast cancer gene 2	DNA damage response	Frameshift deletion	13:g.32911659_32911663del	K1057Tfs*8	Pathogenic (ClinVar ID: 37826)
	NOTCH1	Neurogenic locus notch homolog protein 1	Notch signaling, promotion of differentiation of progenitor cells into astroglia	Missense	9:g.139390828T>C	T2455A	Conflicting interpretations of pathogenicity: Uncertain significance (5) vs. Benign (1) (ClinVar ID: 264349)
	PDGFRA	Platelet‐derived growth factor receptor alpha	Tyrosine kinase receptor for members of the platelet‐derived growth factor family	Missense	4:g.55124981A>T	T16S	Conflicting interpretations of pathogenicity: Uncertain significance (3) vs. Likely benign (1) (ClinVar ID: 135017)
	ASXL1	ASXL transcriptional regulator 1	Regulator of chromatin remodeling	Missense	20:g.31023065A>T	E850D	—
	RIF1	Replication timing regulatory factor 1	Regulator of telomere length, DNA repair	Missense	2:g.152320874A>C	I1614L	—
GENIE‐DFCI‐089908 (primary melanoma and pancreatic adenocarcinoma metastasis)	FANCA	FA Complementation Group A	DNA repair	Missense	16:g.89811368C>T	D1209N	Uncertain significance (ClinVar ID: 2180921)
	SBDS	SBDS ribosome maturation Factor, also known as Shwachman–Bodian–Diamond Syndrome Protein	Ribosome biogenesis	Missense	7:g.66453447C>G	E222Q	Uncertain significance (ClinVar ID: 1163279)
	NOTCH3	Neurogenic locus notch homolog protein 3	Notch signaling, promotion of differentiation of progenitor cells into astroglia	Missense	19:g.15271870G>A	A2190V	Benign/Likely benign (ClinVar ID: 803537)
	PRKAR1A	Protein kinase CAMP‐dependent Type I Regulatory Subunit Alpha	Regulatory subunit of the cAMP‐dependent protein kinase (PKA) complex	Missense	17:g.66524013G>C	E247D	Uncertain significance (ClinVar ID: 1434024)
	FAAP100	FA Core Complex Associated Protein 100	DNA damage response pathway	Missense	17:g.79514704T>G	R468S	—

^a^ HGVS genomic nomenclature indicates the chromosome number, reference sequence (g: genomic), nucleotide position, nucleotide variation (Es. T>G).

In patient GENIE‐DFCI‐202272 (male), both tumors present a frameshift deletion in *BRCA2* gene, namely BRCA2 K1057Tfs*8, which is a truncating mutation in a tumor suppressor gene, and therefore is likely oncogenic. Standards and guidelines for the interpretation of sequence variants have been published and they recommend the use of specific standard terminology “pathogenic,” “likely pathogenic,” “uncertain significance,” “likely benign,” and “benign” to describe variants identified in genes that cause Mendelian disorders (Richards et al. 2015). Moreover, in ClinVar, which is a freely accessible public archive about relationships among variation and human health, this mutation has been described as a pathogenic germline mutation. Specifically, this variation has been associated with breast cancer (Breast Cancer Association Consortium et al. 2021; Darst et al. 2021). *BRCA2*, a tumor suppressor involved in the DNA damage response, is mutated in various cancer types (Andreassen et al. 2021). Germline mutations of *BRCA2* predispose individuals to a high risk of breast and ovarian cancer, and elevated risk of other cancers, including those of the pancreas (Nelson et al. 2013; van Asperen et al. 2005) and melanoma (Johansson et al. 2019). Except for *BRCA2* mutation, the biological significance of all the other mutations is still unknown or with an unclear role.

Both patients present mutation in NOTCH family member, namely *NOTCH1* and *NOTCH3*. For the mutation in *NOTCH1* gene, there are conflicting interpretations of pathogenicity, while for *NOTCH3*, the mutation seems to be benign or likely benign. *NOTCH1* is a transmembrane receptor and transcription factor, and can function as both an oncogene and tumor suppressor. In melanoma, *NOTCH1* promotes melanoma growth, progression (Liu et al. 2006) and angiogenesis (Murtas et al. 2015), while in pancreatic cancer, *NOTCH1* seems to act as tumor suppressor gene, in fact, the loss of *NOTCH1* resulted in increased tumor incidence and progression (Hanlon et al. 2010). *NOTCH3* encodes a Type I transmembrane protein of the NOTCH family, and missense and nonsense mutations in *NOTCH3* have been identified in various cancers. In melanoma, *NOTCH3* activity is associated with enhanced melanoma cell migration, while in pancreatic cancer, high expression of *NOTCH3* is correlated with tumor grade, metastasis, high invasion and poor overall survival rates (Zhou et al. 2016).

*PDGFRA*, a receptor tyrosine kinase, is altered by mutation, chromosomal rearrangement, or amplification in a diverse range of cancers. Functional mutations of *PDGFRA* have been described as activator of MAPK and PI3K/AKT pathway in melanoma (Dai et al. 2020), while in pancreatic cancer, *PDGFRA* is able to predict immune infiltration and survival outcomes, specifically, high *PDGFRA* expression is associated with increased immune infiltration and prolonged overall survival (Wu et al. 2022). In the analyzed patient, the mutation has conflicting interpretations of pathogenicity.

Mutations in *ASXL1* and *RIF1* gene in patient GENIE‐DFCI‐202272 (male) have been not yet described and found in ClinVar. *ASXL1* is a tumor suppressor and epigenetic regulator that affects chromatin remodeling. *ASXL1* is inactivated by mutation in various cancer types, most frequently in myeloid malignancies. However, *ASXL1* results to be highly expressed in metastatic melanoma compared to primary melanoma and associated with poor overall survival in melanoma patients (Chen et al. 2019). In a study of familial pancreatic cancer patients, two possibly deleterious germline variants have been detected in *ASXL1* gene (Kasuga et al. 2022). *RIF1* is one of the DNA damage repair (DDR) genes, and it has been reported to play significant roles in the DNA damage response and replication timing regulation. In pancreatic cancer, mutations in this gene have been reported (Stoof et al. 2021), while in melanoma, this gene has been not yet described.

From the same family of DNA damage repair genes, there is *FANCA* gene. FANCA is a tumor suppressor and DNA repair protein. Germline mutations of *FANCA* have been associated with the cancer predisposition syndrome Fanconi Anemia. Genetic variants in Fanconi anemia pathway genes, namely *BRCA2* and *FANCA*, could predict melanoma survival (Yin et al. 2015). *FANCA* has been also examined as a candidate susceptibility gene for familial pancreatic cancer, but the 12 analyzed germline *FANCA* gene mutations found in 44 patients with familial pancreatic cancer did not contribute to familial pancreatic cancer susceptibility (Rogers et al. 2004). Mutation found in GENIE‐DFCI‐089908 patient has conflicting interpretations of pathogenicity. Another gene linked to Fanconi anemia pathway is *FAAP100* gene. *FAAP100* seems to be essential for activation of the Fanconi anemia‐associated DNA damage response pathway (Ling et al. 2007). The mutation found in GENIE‐DFCI‐089908 patient has never been described in ClinVar and literature about this gene in melanoma and pancreatic cancer is missing.

GENIE‐DFCI‐089908 patient showed other two variations in two genes with uncertain significance, namely in *SBDS*, *PRKAR1A* genes. It was suggesting a role of *SBDS* in the pathogenesis of, or response to, inflammatory and neoplastic pancreatic diseases, including pancreatic cancer (Kayed et al. 2008). *PRKAR1A* encodes for a regulatory subunit of protein kinase A and its mutations are associated with Carney complex syndrome and adrenocortical tumors. Carney complex (CNC) is a rare disease associated with multiple neoplasias, including a predisposition to pancreatic tumors; it is caused most frequently by the inactivation of the *PRKAR1A* gene (Saloustros et al. 2017). In addition, the protein product of *PRKAR1A* gene can be used as a marker for the identification of specific histological subtypes of cutaneous nevi and CM (Ricci et al. 2022).

Although mutations are known as the most commonly genetic modifications able to cause cancer, genomic alterations such as copy number alterations (CNA), also known as copy number variation (CNV), are also playing an emergent role. CNA refer to a phenomenon in which segments of the genome are repeated or deleted, with varying numbers of these repeats among different individuals' genomes.

For this reason, in addition to mutated gene, we investigate and compared genes affected by copy number alteration with a frequency of at least 3% in both melanoma and pancreatic cancer (Table 7).

**TABLE 7:** Common copy number alterations (CNA) in melanoma and pancreatic cancer.

Gene	Description—gene name	Pathway	Cytoband	Copy number alteration	Frequency in melanoma	Profiled samples (*n*) in melanoma	Frequency in pancreatic cancer	Profiled samples (*n*) in pancreatic cancer
CDKN2A	Cyclin‐dependent kinase inhibitor 2A	Cell‐cycle	9p21.3	HOMDEL	22.1%	2916	14.8%	5959
CDKN2B	Cyclin‐dependent kinase inhibitor 2B	Cell‐cycle	9p21.3	HOMDEL	17.5%	2881	13.8%	5874
MTAP	Methylthioadenosine phosphorylase	Methionine salvage pathway	9p21.3	HOMDEL	9.9%	1421	11.0%	2898
CCND1	Cyclin D1	Cell‐cycle	11q13.3	AMP	3.5%	2911	—	—
FGF3	Fibroblast growth factor 3	Negative regulator of bone growth	11q13.3	AMP	3.5%	1844	—	—
FGF4	Fibroblast growth factor 4	Regulator in developmental process.	11q13.3	AMP	3.6%	1746	—	—
FGF19	Fibroblast growth factor 19	Regulator of bile acid synthesis, triglycerides levels, gluconeogenesis, and glycogen and protein synthesis	11q13.3	AMP	3.8%	1844	—	—
GAB2	GRB2‐associated‐binding protein 2	Activator of PI3K pathway	11q14	AMP	3.2%	475	—	—
HSD3B1	Hydroxy‐delta‐5‐steroid dehydrogenase, 3 beta‐ and steroid delta‐isomerase 1	Regulator of production of steroid hormones	1p12	AMP	3.5%	227	—	—
PTEN	Phosphatase and tensin homolog	Regulator of PIP3 signaling	10q23.31	HOMDEL	3.1%	2916	—	—
PTPN6	Protein tyrosine phosphatase non‐receptor type 6	Regulator of PIP3 signaling	12p13.31	AMP	100%	1	—	—
MYC	MYC proto‐oncogene	Regulates the expression of genes involved in cell‐cycle control	8q24.21	AMP	—	—	3.2%	5959
SMAD4	SMAD family member 4	Member of TGF‐β1/SMAD4 signaling pathway	18q21.2	HOMDEL	—	—	4.2%	5960

Abbreviations: AMP, amplification; HOMDEL, homozygous deletion.

In melanoma samples, we found 11 CNA, while in pancreatic cancer, we detected five CNA. The three common CNA in both melanoma and pancreatic cancer are *CDKN2A*, *CDKN2B*, and *MTAP*, which are homozygous deleted and located in the same locus, namely 9p21. Chr9p22.1‐21.3 locus deletions have already been related to the development of several types of cancer, especially due to the presence of *CDKN2A* and *CDKN2B* genes (Goncalves, Reis, and Bidinotto 2022). *CDKN2B* is considered a tumor suppressor and encodes a cyclin‐dependent kinase inhibitor that regulates cell growth and the cell cycle G1 progression by preventing the activation of cyclin‐D‐dependent kinases. In various tumors, *CDKN2B* has been found to be frequently co‐deleted with the neighboring tumor suppressor gene *CDKN2A*. *CDKN2B* deletion has been reported in a significantly high proportion in pancreatic cancer (Oketch, Giulietti, and Piva 2023) and in melanoma (McNeal et al. 2015). In addition of *CDKN2A* and *CDKN2B*, there are several other genes in the region with potential importance in tumorigenesis, such as *MTAP*, which results to be homozygous deleted. In melanoma, the loss of *MTAP* was shown to have an effect on tumor invasion and metastasis. In addition, the loss of MTAP results in an inhibition of STAT signaling pathways regulated by interferon, which to ineffectiveness of interferon therapy (Wild et al. 2007). In pancreatic cancer, it has been observed that MTAP deficiency drives tumor progression by inducing metabolic reprogramming, providing a novel target and therapeutic strategy for treating MTAP‐deficient disease (Hu et al. 2021).

## 5 Conclusion

To date, it is known that familial melanoma and pancreatic cancer can be associated with an inheritance pattern of germline mutations in predisposing genes. This study contributes valuable insights into the genetic underpinnings of melanoma and pancreatic cancer, urging the continued exploration and research of new genetic biomarkers able to identify patients at high risk of developing both cancers. Understanding association between cancer risk and gene mutations is of great help for clinicians involved in cancer screening: It can guide the clinician in performing appropriate genetic testing and surveillance in these families. Mutations in the *CDKN2A* gene present the most prevalent genetic cause of increased susceptibility to the development of both melanoma and pancreatic cancer. Other genes found in literature and in open‐access resources have been described; however, their role driving or predisposing both tumors still need to be investigated. A larger sample of patients and families with both melanoma and pancreatic cancer needs to be studied in order to find new candidates predisposing to the occurrence of these cancers.

All members of families with combined occurrence of pancreatic cancer and melanoma should be counseled and offered screening for *CDKN2A* mutations to identify high‐risk family members who should be enrolled in a clinical screening program. In future, the identification of individuals with a pathogenic gene variant in *CDKN2A* gene or in other genes will hold significance for screening at‐risk relatives and devising an effective clinical strategy for the patient.

## Author Contributions

Conception and design: Elisabetta Broseghini, Federico Venturi, Manuela Ferracin, Emi Dika. Acquisition of data: Elisabetta Broseghini, Federico Venturi. Analysis and interpretation of data: Elisabetta Broseghini, Federico Venturi, Giulia Veronesi, Biagio Scotti, Marina Migliori, Desy Marini, Claudio Ricci, Riccardo Casadei, Manuela Ferracin, Emi Dika. Drafting of the manuscript: Elisabetta Broseghini, Federico Venturi, Emi Dika. Critical revision of the manuscript for important intellectual content: Manuela Ferracin, Emi Dika. All authors reviewed the results and approved the final version of the manuscript.

## Conflicts of Interest

The authors declare no conflicts of interest.

## Supporting information


Table S1.

Table S2.

## Data Availability

The authors have nothing to report.
