# Supplementary material for: Exploring the Common Mutational Landscape in Cutaneous Melanoma and Pancreatic Cancer
Source: Pigment Cell Melanoma Res. 2024 Nov 28;38(1):e13210. doi: 10.1111/pcmr.13210 (PMC11681848; doi:10.1111/pcmr.13210)
Supplement: Supplementary file 1 — Table S1. [file PCMR-38-0-s002.docx]

| Supplementary Table 1 |  | | |
| --- | --- | --- | --- |
| Mutated genes in melanoma samples with a frequency ≥ 5% | | | |
| Gene | **Mutated samples** | **Profiled Samples** | **Frequency** |
| ABCB11 | 189 | 1212 | 15.6% |
| ADGRA2 | 87 | 1403 | 6.2% |
| ADGRB3 | 5 | 57 | 8.8% |
| ADGRL3 | 3 | 57 | 5.3% |
| ALB | 41 | 524 | 7.8% |
| ALK | 555 | 6409 | 8.7% |
| AMER1 | 184 | 3053 | 6.0% |
| ANKRD11 | 245 | 2246 | 10.9% |
| ANKRD24 | 3 | 56 | 5.4% |
| APC | 531 | 6042 | 8.8% |
| APOB | 5 | 53 | 9.4% |
| AR | 254 | 4328 | 5.9% |
| ARHGAP35 | 112 | 1817 | 6.2% |
| ARHGEF12 | 52 | 926 | 5.6% |
| ARID1A | 458 | 4269 | 10.7% |
| ARID1B | 354 | 4004 | 8.8% |
| ARID2 | 586 | 4049 | 14.5% |
| ASXL1 | 294 | 4130 | 7.1% |
| ASXL2 | 187 | 2717 | 6.9% |
| ATM | 473 | 6112 | 7.7% |
| ATP8B1 | 19 | 328 | 5.8% |
| ATR | 331 | 4095 | 8.1% |
| ATRX | 400 | 4240 | 9.4% |
| AXL | 335 | 4239 | 7.9% |
| BCL11B | 128 | 1043 | 12.3% |
| BCLAF1 | 3 | 53 | 5.7% |
| BCOR | 269 | 4111 | 6.5% |
| BCORL1 | 167 | 2618 | 6.4% |
| BLM | 213 | 4049 | 5.3% |
| BOD1L1 | 4 | 56 | 7.1% |
| BRAF | 2461 | 6411 | 38.4% |
| BRCA1 | 232 | 4334 | 5.4% |
| BRCA2 | 467 | 4279 | 10.9% |
| BRD4 | 301 | 4069 | 7.4% |
| BRIP1 | 237 | 4136 | 5.7% |
| CADM2 | 14 | 182 | 7.7% |
| CAMTA1 | 36 | 384 | 9.4% |
| CARD11 | 545 | 4141 | 13.2% |
| CASR | 50 | 341 | 14.7% |
| CBFA2T3 | 57 | 926 | 6.2% |
| CBL | 358 | 4267 | 8.4% |
| CBLB | 105 | 1317 | 8.0% |
| CDH23 | 8 | 63 | 12.7% |
| CDKN2A | 584 | 6222 | 9.4% |
| CHD1 | 23 | 445 | 5.2% |
| CHD2 | 84 | 1421 | 5.9% |
| CHD4 | 75 | 1420 | 5.3% |
| CIC | 325 | 3937 | 8.3% |
| CIITA | 124 | 1200 | 10.3% |
| CREBBP | 370 | 4264 | 8.7% |
| CRTC1 | 62 | 1171 | 5.3% |
| CSF3R | 228 | 3876 | 5.9% |
| CSMD3 | 6 | 111 | 5.4% |
| CTNNB1 | 320 | 6409 | 5.0% |
| CUX1 | 271 | 2315 | 11.7% |
| DDX3X | 30 | 529 | 5.7% |
| DEPDC5 | 12 | 182 | 6.6% |
| DICER1 | 263 | 4023 | 6.5% |
| DIS3L2 | 72 | 1225 | 5.9% |
| DNAH9 | 10 | 60 | 16.7% |
| DNMT1 | 143 | 2654 | 5.4% |
| DOT1L | 224 | 3057 | 7.3% |
| DROSHA | 96 | 1589 | 6.0% |
| DYNC2H1 | 3 | 53 | 5.7% |
| EBF1 | 28 | 515 | 5.4% |
| EGFR | 429 | 6410 | 6.7% |
| EP300 | 275 | 4136 | 6.6% |
| EP400 | 8 | 111 | 7.2% |
| EPHA3 | 395 | 3219 | 12.3% |
| EPHA7 | 515 | 3080 | 16.7% |
| EPHB1 | 213 | 3058 | 7.0% |
| ERBB4 | 891 | 6273 | 14.2% |
| ERCC5 | 172 | 3456 | 5.0% |
| ERG | 261 | 3967 | 6.6% |
| FAAP100 | 46 | 924 | 5.0% |
| FAN1 | 50 | 924 | 5.4% |
| FANCD2 | 166 | 2278 | 7.3% |
| FANCI | 104 | 2062 | 5.0% |
| FANCM | 91 | 1170 | 7.8% |
| FAT1 | 664 | 3829 | 17.3% |
| FGFR2 | 450 | 6410 | 7.0% |
| FGFR4 | 278 | 4352 | 6.4% |
| FLT1 | 581 | 4145 | 14.0% |
| FLT3 | 482 | 6116 | 7.9% |
| FLT4 | 468 | 4054 | 11.5% |
| GABRA6 | 75 | 1075 | 7.0% |
| GLI1 | 278 | 3941 | 7.1% |
| GLI2 | 308 | 1514 | 20.3% |
| GRID1 | 4 | 72 | 5.6% |
| GRIN2A | 718 | 2990 | 24.0% |
| GRM3 | 203 | 1482 | 13.7% |
| H19 | 5 | 53 | 9.4% |
| HABP2 | 65 | 924 | 7.0% |
| HDAC4 | 30 | 513 | 5.8% |
| HGF | 260 | 3052 | 8.5% |
| IGF2R | 44 | 670 | 6.6% |
| IKZF1 | 409 | 4085 | 10.0% |
| IKZF3 | 36 | 674 | 5.3% |
| IL2RB | 9 | 157 | 5.7% |
| IL7R | 467 | 3875 | 12.1% |
| INHBA | 152 | 2919 | 5.2% |
| INSR | 141 | 2520 | 5.6% |
| ITK | 101 | 958 | 10.5% |
| KAT6A | 182 | 2381 | 7.6% |
| KDM5A | 224 | 3904 | 5.7% |
| KDM6B | 21 | 246 | 8.5% |
| KDR | 673 | 6112 | 11.0% |
| KEL | 113 | 1077 | 10.5% |
| KIF1B | 88 | 937 | 9.4% |
| KIT | 339 | 6411 | 5.3% |
| KMT2A | 528 | 4160 | 12.7% |
| KMT2B | 455 | 2516 | 18.1% |
| KMT2C | 436 | 2853 | 15.3% |
| KMT2D | 931 | 4110 | 22.7% |
| KNSTRN | 93 | 1838 | 5.1% |
| LAMB4 | 4 | 72 | 5.6% |
| LIFR | 24 | 234 | 10.3% |
| LRP1B | 510 | 1470 | 34.7% |
| MAGI2 | 96 | 1013 | 9.5% |
| MAP2K1 | 265 | 4551 | 5.8% |
| MAP3K1 | 206 | 4116 | 5.0% |
| MAP3K4 | 40 | 783 | 5.1% |
| MAP3K5 | 29 | 449 | 6.5% |
| MDC1 | 223 | 2312 | 9.6% |
| MECOM | 282 | 1148 | 24.6% |
| MED12 | 244 | 4123 | 5.9% |
| MET | 498 | 6392 | 7.8% |
| MGA | 435 | 3535 | 12.3% |
| MST1R | 158 | 2511 | 6.3% |
| MTA1 | 51 | 924 | 5.5% |
| MTOR | 369 | 4470 | 8.3% |
| MYH9 | 34 | 522 | 6.5% |
| NCOA2 | 36 | 518 | 6.9% |
| NCOA3 | 168 | 2705 | 6.2% |
| NCOR1 | 193 | 2959 | 6.5% |
| NCOR2 | 8 | 124 | 6.5% |
| NECTIN4 | 64 | 924 | 6.9% |
| NF1 | 945 | 4336 | 21.8% |
| NFKBIE | 105 | 1329 | 7.9% |
| NIN | 9 | 113 | 8.0% |
| NIPBL | 39 | 477 | 8.2% |
| NOTCH1 | 409 | 6248 | 6.5% |
| NOTCH2 | 310 | 4152 | 7.5% |
| NOTCH3 | 639 | 4071 | 15.7% |
| NOTCH4 | 378 | 2576 | 14.7% |
| NRAS | 1591 | 6411 | 24.8% |
| NRG1 | 189 | 2047 | 9.2% |
| NRIP1 | 17 | 328 | 5.2% |
| NSD1 | 302 | 3842 | 7.9% |
| NSD2 | 146 | 2740 | 5.3% |
| NTRK1 | 313 | 4351 | 7.2% |
| NTRK3 | 506 | 4244 | 11.9% |
| NUTM1 | 145 | 1312 | 11.1% |
| OR5L1 | 11 | 157 | 7.0% |
| PAK5 | 498 | 2487 | 20.0% |
| PAX7 | 64 | 1274 | 5.0% |
| PAXIP1 | 50 | 924 | 5.4% |
| PBRM1 | 255 | 4079 | 6.3% |
| PCDHAC2 | 3 | 56 | 5.4% |
| PDE4DIP | 7 | 113 | 6.2% |
| PDGFRA | 434 | 6176 | 7.0% |
| PDGFRB | 329 | 4269 | 7.7% |
| PGR | 159 | 2246 | 7.1% |
| PIK3C2B | 154 | 2152 | 7.2% |
| PIK3C2G | 425 | 2549 | 16.7% |
| PIK3CG | 293 | 3021 | 9.7% |
| PKD1L2 | 6 | 56 | 10.7% |
| PKHD1 | 8 | 116 | 6.9% |
| PLCG2 | 188 | 2495 | 7.5% |
| POLD1 | 227 | 3936 | 5.8% |
| POLE | 395 | 4695 | 8.4% |
| POLQ | 145 | 1052 | 13.8% |
| POLR2A | 24 | 328 | 7.3% |
| PPARG | 193 | 3675 | 5.3% |
| PPP6C | 152 | 2680 | 5.7% |
| PRDM1 | 291 | 4111 | 7.1% |
| PRDM14 | 83 | 1301 | 6.4% |
| PREX2 | 648 | 2697 | 24.0% |
| PRF1 | 65 | 1132 | 5.7% |
| PRKDC | 366 | 2526 | 14.5% |
| PRKN | 215 | 4065 | 5.3% |
| PRPF8 | 38 | 534 | 7.1% |
| PRSS1 | 94 | 1140 | 8.2% |
| PTCH1 | 235 | 4273 | 5.5% |
| PTCH2 | 36 | 612 | 5.9% |
| PTEN | 458 | 6356 | 7.2% |
| PTK2B | 104 | 1041 | 10.0% |
| PTPN14 | 57 | 924 | 6.2% |
| PTPRB | 107 | 445 | 24.0% |
| PTPRD | 675 | 3097 | 21.8% |
| PTPRS | 195 | 2312 | 8.4% |
| PTPRT | 823 | 2799 | 29.4% |
| RAC1 | 213 | 4282 | 5.0% |
| RAD54B | 49 | 924 | 5.3% |
| RANBP2 | 104 | 1015 | 10.2% |
| RASA2 | 39 | 499 | 7.8% |
| RBBP8 | 49 | 924 | 5.3% |
| RET | 404 | 6409 | 6.3% |
| RICTOR | 264 | 4094 | 6.4% |
| RIF1 | 108 | 924 | 11.7% |
| ROS1 | 1007 | 5022 | 20.1% |
| RPTOR | 258 | 3941 | 6.5% |
| RUNX1T1 | 258 | 2349 | 11.0% |
| SAMD9 | 28 | 399 | 7.0% |
| SERPINB3 | 28 | 515 | 5.4% |
| SERPINB4 | 29 | 515 | 5.6% |
| SETBP1 | 304 | 2365 | 12.9% |
| SETD2 | 458 | 4239 | 10.8% |
| SF3B1 | 294 | 4371 | 6.7% |
| SIN3A | 24 | 445 | 5.4% |
| SLC26A3 | 3 | 53 | 5.7% |
| SLC34A2 | 89 | 1016 | 8.8% |
| SLFN11 | 41 | 515 | 8.0% |
| SLIT2 | 245 | 1420 | 17.3% |
| SLITRK6 | 23 | 299 | 7.7% |
| SLX4 | 321 | 3094 | 10.4% |
| SMARCA2 | 48 | 920 | 5.2% |
| SMARCA4 | 425 | 4244 | 10.0% |
| SMARCAL1 | 24 | 328 | 7.3% |
| SNCAIP | 252 | 1469 | 17.2% |
| SPEN | 361 | 3052 | 11.8% |
| SPTA1 | 352 | 1407 | 25.0% |
| SYK | 184 | 3226 | 5.7% |
| SYNE1 | 71 | 972 | 7.3% |
| TBX3 | 162 | 3033 | 5.3% |
| TEK | 189 | 1800 | 10.5% |
| TERT | 2076 | 4129 | 50.3% |
| TET1 | 268 | 3269 | 8.2% |
| TET2 | 545 | 4164 | 13.1% |
| TGFB1 | 4 | 56 | 7.1% |
| THADA | 27 | 382 | 7.1% |
| TLR2 | 4 | 56 | 7.1% |
| TLR4 | 49 | 370 | 13.2% |
| TNK2 | 7 | 111 | 6.3% |
| TOPBP1 | 66 | 956 | 6.9% |
| TP53 | 1194 | 6356 | 18.8% |
| TP53BP1 | 168 | 2366 | 7.1% |
| TP63 | 390 | 2381 | 16.4% |
| TRIM37 | 47 | 924 | 5.1% |
| TSC2 | 328 | 4282 | 7.7% |
| TSHR | 202 | 3982 | 5.1% |
| TSHZ3 | 18 | 157 | 11.5% |
| TYK2 | 43 | 516 | 8.3% |
| UBR5 | 8 | 145 | 5.5% |
| USP28 | 59 | 924 | 6.4% |
| USP6 | 43 | 416 | 10.3% |
| USP7 | 9 | 157 | 5.7% |
| WAS | 52 | 1029 | 5.1% |
| WNK2 | 4 | 51 | 7.8% |
| WRN | 123 | 1621 | 7.6% |
| WT1 | 205 | 4139 | 5.0% |
| ZFHX3 | 349 | 2664 | 13.1% |
| ZNRF3 | 83 | 1561 | 5.3% |
