# Supplementary material for: Exploring the Common Mutational Landscape in Cutaneous Melanoma and Pancreatic Cancer
Source: Pigment Cell Melanoma Res. 2024 Nov 28;38(1):e13210. doi: 10.1111/pcmr.13210 (PMC11681848; doi:10.1111/pcmr.13210)
Supplement: Supplementary file 2 — Table S2. [file PCMR-38-0-s001.docx]

| Supplementary Table 2 |  | | |
| --- | --- | --- | --- |
| Mutated genes in pancreatic cancer samples with a frequency ≥ 5% | | | |
| Gene | **Mutated samples** | **Profiled Samples** | **Frequency** |
| ADGRL3 | 3 | 55 | 5.5% |
| ANKRD24 | 7 | 54 | 13.0% |
| ARFGAP3 | 4 | 54 | 7.4% |
| ARID1A | 606 | 7303 | 8.3% |
| BOD1L1 | 6 | 54 | 11.1% |
| CACNA1E | 4 | 54 | 7.4% |
| CDH23 | 12 | 77 | 15.6% |
| CDKN2A | 755 | 8041 | 9.4% |
| COL7A1 | 101 | 1435 | 7.0% |
| DNAH9 | 15 | 84 | 17.9% |
| DOCK8 | 72 | 1435 | 5.0% |
| KDM6B | 22 | 410 | 5.4% |
| KMT2D | 500 | 7127 | 7.0% |
| KRAS | 6372 | 8073 | 78.9% |
| LAMB4 | 8 | 147 | 5.4% |
| LRP1B | 114 | 1267 | 9.0% |
| PDE4DIP | 12 | 209 | 5.7% |
| PKD1L2 | 8 | 54 | 14.8% |
| PRKDC | 160 | 2934 | 5.5% |
| RANBP2 | 52 | 958 | 5.4% |
| RNF213 | 15 | 209 | 7.2% |
| RNF43 | 426 | 7205 | 5.9% |
| SMAD4 | 1464 | 8042 | 18.2% |
| SPTA1 | 69 | 1191 | 5.8% |
| TGFB1 | 3 | 54 | 5.6% |
| TP53 | 5151 | 8046 | 64.0% |
| UMODL1 | 4 | 54 | 7.4% |
